# Supplementary material for: Novel Cytoskeleton-Associated Proteins in Trypanosoma brucei Are Essential for Cell Morphogenesis and Cytokinesis
Source: Microorganisms. 2021 Oct 27;9(11):2234. doi: 10.3390/microorganisms9112234 (PMC8625193; doi:10.3390/microorganisms9112234)
Supplement: Supplementary file 1 [file microorganisms-09-02234-s001.zip › microorganisms-1421795-supplementary.pdf]

Supplementary table 1: DNA oligonucleotides used in this study

**Name**	**Purpose, Restriction site**	**Sequence**
pHD BirA for	BirA* amplification, *Asc*I	ATAGGGCGCGCCGAACAAAAACTCATCTCAGAAG
pHD BirA rev	BirA* amplification, *Pac*I	TATTTAATTAAGTCGAGCTTCTCTGCGCTTCTC
CAP5.5ORFfor	CAP5.5 ORF amplification, *Fse*I	ATAGGCCGGCCATGGGTTGTGGTGGATCAAAG
CAP5.5ORFrev	CAP5.5 ORF amplification, *Asc*I	TATGGCGCGCCCATCACTGTCAGTTTCATCAC
Tb927.6.5070_RNAi_for	CAP52 RNAi, *Bam*HI, *Hind*III	ATAGGATCCAAGCTTCTGTTCTTGCAATGAGC
Tb927.6.5070_RNAi_rev	CAP52 RNAi, *Xho*I, *Xba*I	ATACTCGAGTCTAGAGGAACTCTTGAGCCTTC
Tb927.4.1300_RNAi_for	CAP42 RNAi, *Bam*HI, *Hind*III	ATAGGATCCAAGCTTATGTCGACTGCTCTGTTCG
Tb927.4.1300_RNAi_rev	CAP42 RNAi, *Xho*I, *Xba*I	ATACTCGAGTCTAGATGACAACCATAATGTTG
Tb927.11.2610RNAi2_for	CAP50 RNAi, *Bam*HI, *Hind*III	ATAGGATCCAAGCTTGTACGGAGAAAGAAATCACC
Tb927.11.2610RNAi2_rev	CAP50 RNAi, *Xho*I, *Xba*I	ATACTCGAGTCTAGACCTGCTGGCGAGGCGGTCAACAGC
Tb927.6.5070_ORF_for	CAP52 ORF amplification, *Fse*I	ATAGGCCGGCCATGCGAGCAAACCCAATTGAGCG
Tb927.6.5070_ORF_rev	CAP52 ORF amplification, *Asc*I	TATGGCGCGCCCATACGCCCTCCACAGTGGACC
Tb927.4.1300_ORF_for	CAP42 ORF amplification, *Fse*I	ATAGGCCGGCCATGTCGACTGCTCTGTTCGTTC
Tb927.4.1300_ORF_rev	CAP42 ORF amplification, *Asc*I	TATGGCGCGCCCGCAACCGCCGTCCTTAATGTC
Tb927.11.2610_ORF_for	CAP50 ORF and ΔC amplification, *Fse*I	ATAGGCCGGCCATGGCAGCCGCTGTTACAATTG
Tb927.11.2610_ORF_rev	CAP50 ORF and ΔN amplification, *Asc*I	TATGGCGCGCCCCGACGTGCGTTCGACTTGGGTG
Tb927.11.2610ΔC_rev	CAP50ΔN amplification, *Asc*I	TATGGCGCGCCCATCAATTTGGTCAAAAGCGATTTCAGC
Tb927.11.2610ΔN_for	CAP50ΔC amplification, *Fse*I	ATAGGCCGGCCATGCGTAATGGACCCCGCATC
PFRAqPCRf	qPCR	CGTTGGAGATGTTTGGACCT
PFRAqPCRr	qPCR	GCACGGTACTCCACCATCTT
1300q_for	qPCR	CGGTTGGAGGAGGATGCTGG
1300q_rev	qPCR	GTATTGACATGGAGTCACCGACA
5070q_for	qPCR	GATTCCAGTGGAGAGCCTGGGAAG
5070q_rev	qPCR	GCTTCCACGCCAATGGTGCG
Tb927.11.2610qfor	qPCR	GGAGGTTGAGGAACAGCACG
Tb927.11.2610qrev	qPCR	CTCCATCTCGGCGGGCAGTT

Supplementary Figure 1

A)

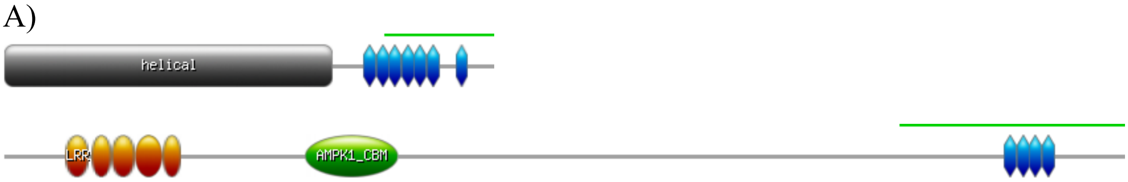

B)

```
CAP50(321-441)       VKKVRRIRRVKRPTKKAEEPAE---------GEAAAEGEAPKEGEAAAEGEAPKEGEAAQ
TbAIR9(853-999)      AVTTAEIAAAELGAAGGEEDAEKPTDQRGRSTEAAADEDAEKSTEAAADEGAEKSTEAAA

CAP50(321-441)       GGEAPKEGEAAQGGEAPKEGEAAQGGEAPKEGEAAQGGEAAVE----------------K
TbAIR9(853-999)      DEGAEKSTEAAADEGAEKSTEAAADEDAEKPTDANPSAEAAVRLDASSTDETSTSAPAPM

CAP50(321-441)       PQGAQVTEGQEN-VKEPEAAATGENGAG
TbAIR9(853-999)      PGAAQALAAALNSVTSSAASTTRTDGA
```

Sequence Analysis of CAP50 and AIR9. A) Domain organization. CAP50 contains a N-terminal α-helical region (grey box). Several repeats can be detected in the C-terminal, unstructured region (green bar). In AIR9, Leucin-Rich repeats (LRR, orange) and a AMP-activated protein kinase (green) can be identified in the N-terminal region, followed by longer, structured repeats (not shown here). Again, repeats can be identified in the intrinsically unstructured region at the C-terminus. B) Sequence alignment of the two repeat-containing regions of CAP50 and TbAIR9. The individual repeats are highlighted. The common EAAA-motif is underlined.

Method: Sequence Analysis

NCBI BLAST have been used to identify similarities to other sequences using the UniProt database. Domain analysis were performed, using SMART (Letunic et al. (2020) Nucleic Acids Res doi: 10.1093/nar/gkaa937), prediction of intrinsically unstructured regions using IUPRED2 (Mészáros et al. 2008, doi.org/10.1093/nar/gky384). Repeats have been identified using EMBOSS dotplot (Rice et al 2010, 10.1016/s0168-9525(00)02024-2) & HHRepID (Zimmermann, et al. 2018, doi.org/10.1016/j.jmb.2017.12.007).

Supplementary Figure 2

```
>CAP52_Tb927.6.5070
MRANPIERRKERETALSVRQRELEELRQRKAQLGEELGMELSALEGDNLAAAFPVIQYCGSRPKKDAKKIPVESLGSVMN
QFEIAIKAISQNNRDIEQQITDLNRTIGVEAQRFTKLKRHSKELADATGVSLDPNAVQHLAGKSRDGEDCSGGLKELEET
TVVLEERKALVEKEIRAARQLVKKKEEAVLAMSSALESRQEEIDQLNRLYNDIRVVDRDIKCEKETLREIISEHDIVDTK
LNEAIERNVSRTRLLIEQGINEIKTEIADSVSVSRRGQERVMKAQEFRIDQLEKRLDCINKALKNNHLTRDVEAIVSHKW
AAAGDALVAATPDESMYDIEAIIPPQERCHPAIYNLLLTEKERLARRISLLGIIAKEKKEVIDALACKAEALARECQQAI
QELDHVASAAAYEEEMQRVEAMEYIQKQRLHYSDLFKEMWKLKTKNQGPLWRAY

>Tb927.9.11540
MADVSADSDAQVELERLNDVIDKYTCQVEHIDNLLQELEEENNSDSVSRQIAEYQSALESHPENIPAEDALEVITRLENT
LKIVQRRNHLLEKENGTQNRLLEERSNVLLNATKTFDHIVDVTGWHDKFLFDAEDLRSKVADIREMSNIEAVVQKELRVA
QGIIKKKEAALRQLEELVEQGKEQEAVLNNVYNDIRVKERDCSEVEMQLVRLRKSVAKTDEALAVFDLHNQNASLAYMES
DRDYLRDSVAEMKSTTRRQDNVIKAQLTRQQQLQTRLDVIMKSLREMKLDKKYERNIPKSALVPSASREEPEDVSKILPE
SECIPVPTYRLLHKNNEMLRVIVMRKNMLVLEKNAVIEALEAGLAKYGSALITTYKEQQDLRQNKDMELIELMDDLQQQH
SNYLEKLEELRLQNAALKKKMYRSTRQHAPLKGTRPMR
```

```
CAP52               1 MRANPIERRKERETALSVRQRELEELR----QRKAQLGEELGMELSALEG     46
                               ....:|.|..|.|||.|.    :...|: |.:...|..||.
Tb927.9.11540       1 ---------MADVSADSDAQVELERLNDVIDKYTCQV-EHIDNLLQELEE     40

CAP52              47 DNL--AAAFPVIQYCGSRPKKDAKKIPVESLGSVMNQFEIAIKAISQNNR     94
                      :|.  :.:..:.:| .|..:...:.||.|....|:.:.|..:|.:.:.|.
Tb927.9.11540      41 ENNSDSVSRQIAEY-QSALESHPENIPAEDALEVITRLENTLKIVQRRNH     89

CAP52              95 DIEQQITDLNRTIGVEAQRFTKLKRHSKELADATGVSLDPNAVQHLAGKS    144
                      .:|::....||.          |:..|..|.:||      ....|:...:
Tb927.9.11540      90 LLEKENGTQNRL----------LEERSNVLLNAT------KTFDHIVDVT    123

CAP52             145 R-------DGEDCSGGLKELEETTVVLEERKALVEKEIRAARQLVKKKEE    187
                      .       |.||....:.::.|.:.:    :|:|:||:|.|:.::||||.
Tb927.9.11540     124 GWHDKFLFDAEDLRSKVADIREMSNI----EAVVQKELRVAQGIIKKKEA    169

CAP52             188 AVLAMSSALESRQEEIDQLNRLYNDIRVVDRD---IKCEKETLREIISEH    234
                      |:..:...:|..:|:...||.:||||||.:||   ::.:...||:.::  
Tb927.9.11540     170 ALRQLEELVEQGKEQEAVLNNVYNDIRVKERDCSEVEMQLVRLRKSVA--    217

CAP52             235 DIVDTKLNEAIE----RNVSRTRLLIEQGINEIKTEIADSVSVSRRGQER    280
                           |.:||:.    .|.:.:...:|...:.::..:|:..|.:|| |:.
Tb927.9.11540     218 -----KTDEALAVFDLHNQNASLAYMESDRDYLRDSVAEMKSTTRR-QDN    261

CAP52             281 VMKAQEFRIDQLEKRLDCINKALKNNHLTRDVEAIVSHKWAAAGDALVAA    330
                      |:|||..|..||:.|||.|.|:|:...|.:..|..:..      .|||.:
Tb927.9.11540     262 VIKAQLTRQQQLQTRLDVIMKSLREMKLDKKYERNIPK------SALVPS    305

CAP52             331 TPDESMYDIEAIIPPQERCHPA-IYNLLLTEKERLARRISLLGIIAKEKK    379
                      ...|...|:..|:|..| |.|. .|.||....|.|...:....::..||.
Tb927.9.11540     306 ASREEPEDVSKILPESE-CIPVPTYRLLHKNNEMLRVIVMRKNMLVLEKN    354

CAP52             380 EVIDALACKAEALAR---------ECQQAIQELDHVASAAAYEEEMQRVE    420
                      .||:||..   .||:         :.||.:::          .::|:.:|
Tb927.9.11540     355 AVIEALEA---GLAKYGSALITTYKEQQDLRQ----------NKDMELIE    391

CAP52             421 AMEYIQKQRLHYSDLFKEMWKLKTKNQGPLWRAY----------------    454
                      .|:.:|:|..:|.:..:|   |:.:|.....:.|                
Tb927.9.11540     392 LMDDLQQQHSNYLEKLEE---LRLQNAALKKKMYRSTRQHAPLKGTRPMR    438
```

Pairwise alignment of CAP52 with Tb927.9.11540 using EMBOSS Needle (www.ebi.ac.uk/Tools). Sequences are 24% identical and 42.2% similar.

Supplementary Figure 3

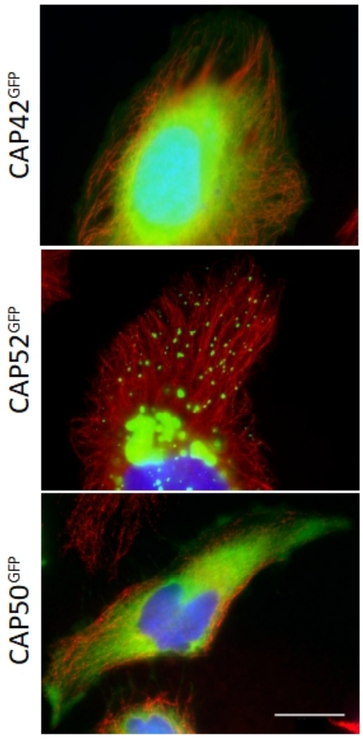

Expression of GFP-tagged CAP42, CAP52 and CAP50 in HeLa cells showed no co-localization of the proteins with mammalian microtubules. Cells were stained with anti-tubulin (red), anti-GFP (green) and the DNA was stained with DAPI. Scale bar = 20 µm
